# Structural variation and evolution of chloroplast *tRNAs* in green algae

**DOI:** 10.7717/peerj.11524

**Published:** 2021-06-01

**Authors:** Fangbing Qi, Yajing Zhao, Ningbo Zhao, Kai Wang, Zhonghu Li, Yingjuan Wang

**Affiliations:** State Key Laboratory of Biotechnology of Shannxi Province, Key Laboratory of Resource Biology and Biotechnology in Western China (Ministry of Education), College of Life Science, Northwest University, Xi’an, China

**Keywords:** Chlorophyceae, Chloroplast *tRNA*, Anticodon, Intron, Evolution

## Abstract

As one of the important groups of the core Chlorophyta (Green algae), Chlorophyceae plays an important role in the evolution of plants. As a carrier of amino acids, *tRNA* plays an indispensable role in life activities. However, the structural variation of chloroplast *tRNA* and its evolutionary characteristics in Chlorophyta species have not been well studied. In this study, we analyzed the chloroplast genome *tRNAs* of 14 species in five categories in the green algae. We found that the number of chloroplasts *tRNAs* of Chlorophyceae is maintained between 28–32, and the length of the gene sequence ranges from 71 nt to 91 nt. There are 23–27 anticodon types of *tRNAs*, and some *tRNAs* have missing anticodons that are compensated for by other types of anticodons of that *tRNA*. In addition, three *tRNAs* were found to contain introns in the anti-codon loop of the *tRNA*, but the analysis scored poorly and it is presumed that these introns are not functional. After multiple sequence alignment, the Ψ-loop is the most conserved structural unit in the *tRNA* secondary structure, containing mostly U-U-C-x-A-x-U conserved sequences. The number of transitions in *tRNA* is higher than the number of transversions. In the replication loss analysis, it was found that green algal chloroplast *tRNAs* may have undergone substantial gene loss during the course of evolution. Based on the constructed phylogenetic tree, mutations were found to accompany the evolution of the Green algae chloroplast *tRNA*. Moreover, chloroplast *tRNAs* of Chlorophyceae are consistent with those of monocotyledons and gymnosperms in terms of evolutionary patterns, sharing a common multi-phylogenetic pattern and rooted in a rich common ancestor. Sequence alignment and systematic analysis of *tRNA* in chloroplast genome of Chlorophyceae, clarified the characteristics and rules of *tRNA* changes, which will promote the evolutionary relationship of *tRNA* and the origin and evolution of chloroplast.

## Introduction

As autotrophic lineages with primary plastids, Viridiplantae, Rhodophyta and Cyanobacteria all originated from an endosymbiotic event, which also marked the origin of oxidative photosynthesis in eukaryotes (Keeling, 2010; Leliaert, Verbruggen & Zechman, 2011). Chlorophyta and Streptophyta are two major lineages that split early in the evolution of green plant (O’Kelly, 2007). The two taxa have subsequently produced the widespread and diverse groups. Among them, green algae in different environments have always maintained relatively independent evolution, not only shows obvious differences in biological characteristics, but also has great differences in genome genetic characteristics (Barsanti & Gualtieri, 2006). It is generally accepted that the Streptophyta branched out into the Land plants and Charophyta. The Chlorophyta gradually formed the extremely widespread core Chlorophyta and other green algae (Leliaert et al., 2012).

The core Chlorophyta including Ulvophyceae, Trebouxiophyceae, Chlorophyceae (UTC) and two small groups namely Chlorodendrophyceae and Pedinophyceae (Fučíková et al., 2014). In the core Chlorophyta, the evolutionary relationships between the various branches and between the UTC branches have been increasingly studied (Lemieux et al., 2015; Leliaert et al., 2016). Chlorodendrophyceae, as a small group of the core Chlorophyceae, is traditionally attributed to prasinophyceae and is considered to be an earlier branch of the core Chlorophyceae. Based on 18s *rDNA* data, a close relationship between Chlorodendrophyceae and UTC is confirmed (Cocquyt et al., 2010). As the main branch of the core Chlorophyta, UTC has abundant species, diverse living environments and the complicated evolutionary relationships. The monophyletic origin of the Chlorophyceae has been confirmed by analysis of a large number of genome sequences (Luo et al., 2010).

Because of the maternal genetic characteristics of chloroplasts, the chloroplast genome is not only structurally stable, but also relatively conservative in number, composition and arrangement (Palmer & Genetics, 1985; Raubeson & Jansen, 2005; Fučíková, Lewis & Lewis, 2016). Thus, studying the evolutionary relationships of species taxa from the perspective of the chloroplast genome can compensate for the deficiencies caused by the analysis of data such as 18S as well as 26S *rDNA*.

**Figure 1:**
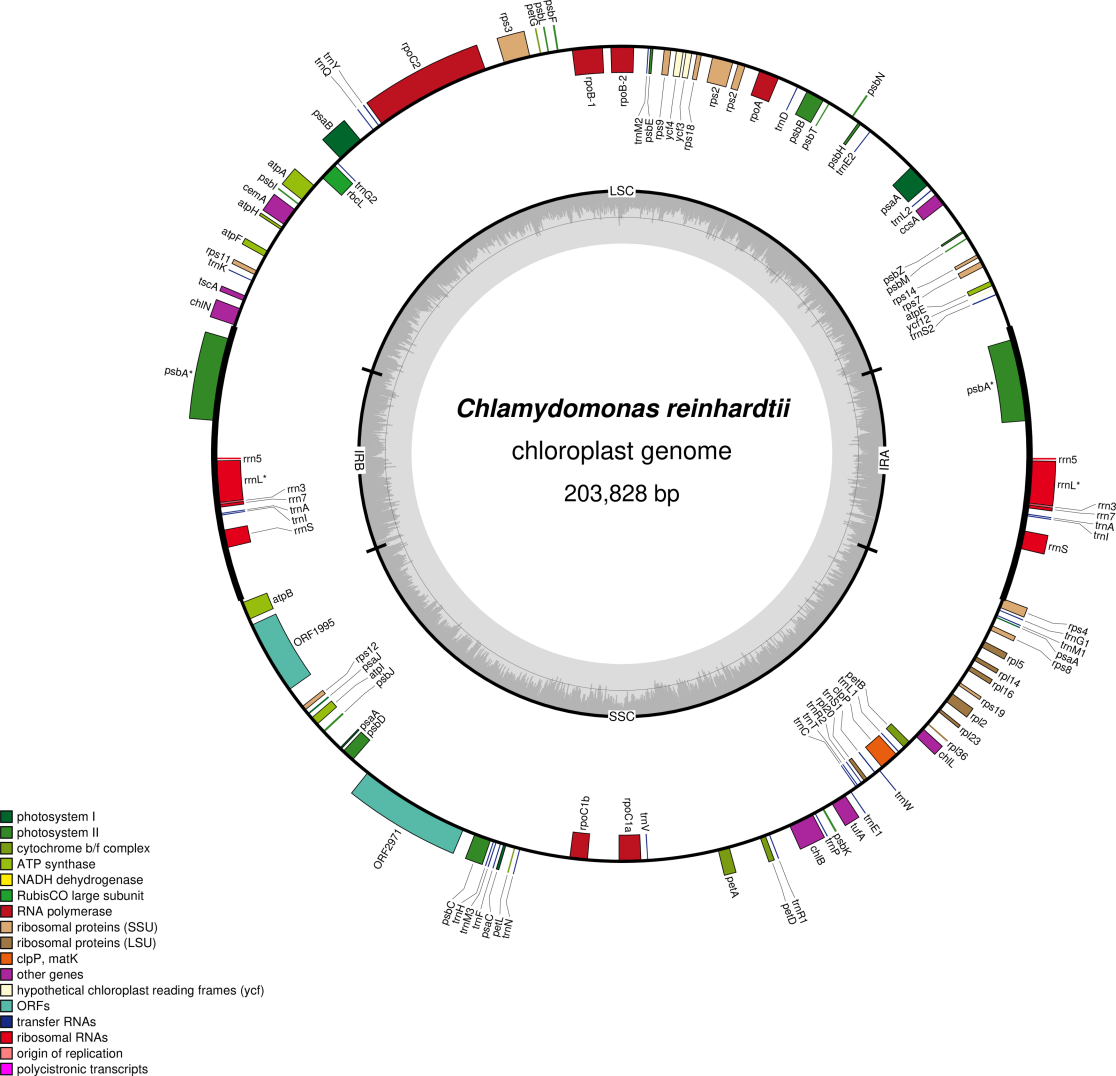
The chloroplast genome of *C. reinhardtii*. The 203828bp genome contains 109 unique genes, including 10 ribosomal *RNA* genes, 29 transfer *RNA* genes, and 69 protein-coding genes. The annotated genes are colored according to the functional categories shown in the legend at the lower left.

The chloroplast genome structure is usually circular, rarely linear, and consists of four parts, the large single copy region (LSC), the small single copy region (SSC) and the inverted repeat regions A and B (IRA, IRB) (Zhang et al., 2012). In most of angiosperm chloroplast genomes, there are generally about 120 coding genes, of which about 80 genes encode proteins involved in photosynthesis and gene expression, another 30 or so genes encode *tRNAs* (Greiner, Lehwark & Bock, 2019) and a few genes encode *rRNAs* (Masanori et al., 2003; Gao, Ying-Juan & Wang, 2010) (Fig. 1).

*tRNA* is essential for linking and matching *mRNA* and amino acids during protein synthesis and is functionally stable. The number of nucleotides of *tRNA* is also relatively conservative, usually 75–95 nt nucleotides (Goodenbour & Pan, 2006). Further, the clover-like secondary structure formed by nucleotide pairing and the inverted L-like tertiary structure formed by further folding have been relatively stable throughout evolution (Holley et al., 1965). So, it has been highly conserved in genetic evolution. Finally, based on the distribution of *tRNAs* in the eukaryotic chloroplast genome, little is known about the structure and its evolutionary mechanisms. We selected chloroplast *tRNAs* from green algae to investigate the structural variation and evolutionary features of chloroplast *tRNAs*.

In our study, we analyzed 14 species from five orders of the core Chlorophyceae to infer the possible evolutionary relationships of *tRNAs* in the chloroplast genome of Chlorophyceae. From the analysis results, we have not only obtained the sequence characteristics and structural features of *tRNAs* in the chloroplast genome of Chlorophyceae, but also discovered that chloroplast *tRNAs* of Chlorophyceae are consistent with those of monocotyledons and gymnosperms in terms of evolutionary patterns, sharing a common multi-phylogenetic pattern and rooted in a multiple common ancestor.

## Materials & Methods

### Sequence analysis of chloroplast *tRNA* genome

We selected 14 representative species from the NCBI genome database in five classes of the core green algae order. They are as following: *Chlamydomonas reinhardtii* (NC_005353.1), *Hafniomonas laevis* (NC_028583.1), *Gonium pectoral* (NC_020438.1), *Characiochloris acuminata* (NC_028584.1), *Phacotus lenticularis* (NC_028587.1) in the volvocales; *Mychonastes homosphaera* (NC_029671.1), *Neochloris aquatica* (NC_029670.1), *Bracteacoccus giganteas* (NC_028586.1), *Tetradesmus obliquus* (NC_008101.1), *Ankyra judayi* (NC_029735.1) in the sphaeropleales; *Schizomeris leibleinii* (NC_015645.1), *Stigeoclonium helveticum* (NC_008372.1) in the chaetophorales; *Oedogonium cardiacum* (NC_011031.1) in the oedogoniales and *Floydiella terrestris* (NC_014346.1) in the chaetopeltidales. We then used tRNAscan-SE software (http://lowelab.ucsc.edu/tRNAscan-SE/) to analyze the whole genome sequences of chloroplasts from different species (Chan et al., 2019). The specific parameters set by the tRNAscan-SE software are: sequence source: mixed (general *tRNA* model); Search mode: default; Query sequence: formatted (FASTA); Genetic Code for *tRNA* Isotype Prediction: universal. From the output of tRNAscan-SE, we can get the overall statistical information as well as the summary data of the whole search (Chan & Lowe, 2019). Through the analysis of the data, the total number of *tRNAs* in the chloroplast genome of each species, the distribution of anticodons of the chloroplast *tRNA*, the number of *tRNAs* with introns, and the gene sequence length of each subtype *tRNA* were recorded.

### Multiple sequence alignment

We grouped the *tRNA* nucleotide sequences of 20 isoforms sequentially and performed *tRNA* isoform multiple sequence alignment using Multalin program to analyze the conserved *tRNA* isoform nucleotide sequences. Multalin’s parameter settings: Sequence input format: Auto, Result page format: a coloured image, Symbol comparison Table—Gap open def.—Gap ext def.: Blosum62-12-2, Gap penalty at opening: default, Gap penalty at extension: default, Gap penalty at extremities: none, One iteration only: no, Text size (image only): MediumBold, Text colour (image only): black, Background colour: white, High consensus colour: red, Low consensus colour: blue, Neutral colour: black, High consensus value: 90%, Low consensus value: 50%, Output style: Normal, Help Maximum line length: 130, Help Graduation step: 10 (Mohanta et al., 2017).

### Phylogenetic tree construction

We constructed a phylogenetic tree based on the genomic sequences of *tRNAs* using MEGA X. Clustal format files of all *tRNAs* were then created by Clustal X 2.0 software. We used MEGA X software to convert the generated *tRNA* Clustal files to MEGA format. For the model selection for constructing a phylogenetic tree, the specific parameters are as follows: Models: Find Best *DNA*/Protein Models (ML); Select a Genetic Code: Standard; Analysis: Tree to Use: Automatic (Neighbor-joining tree), Statistical Method: Maximum likelihood: Substituton Model: Substitutions Type Nucleotide; Data Subset To USE: gaps/Missing Data Treatment: Partial deletion, Site Coverage Cutff (%): 95, Branch Swap Filter: very Strong; System Resource Usage: number of Threads: 3. Among the results of the resulting analysis, we selected the model with the lowest BIC index to construct the phylogenetic tree. Then we used the Phylogeny function to construct the developmental tree, the specific parameters are as follows: Analysis: Statistical Method: Maximum likelihood; Phylogeny test: Bootstrap method, No. of Bootstrap Replications: 1000; Model: Substitutions Type: Nucleotide; Model/Method: kimura-2-parameter model; Rates and patterns: rates among sites: gamma distributed with invariant sites (G+I), no of discrete gamma categories: 5 (Kimura, 1980).

### *tRNA* transition /transversion analysis

We used the *tRNA* files in MEGA format in the MEGA software which further analyzed the transition /transversion rate of all *tRNAs* (Tamura, Nei & Kumar, 2004; Sudhir et al., 2018). The specific analysis process and parameters are as follows: Models: Compute MCL Transition/Transversion Bias, Scope: All Selected Taxa, Statistical Method: Maximum Composite Likelihood, Substitutions Type: Nucleotide, Model/Method: Tamura-Nei model, Gaps/Missing Data Treatment: Pairwise deletion.

### Gene loss and duplication analysis

We analyzed chloroplast genomic *tRNA* gene duplication/loss events in chlorophyceae by Notung2.9 software (Chen, Durand & Farach-Colton, 2000; Vernot et al., 2008). We then used NCBI (https://www.ncbi.nlm.nih.gov/Taxonomy/CommonTree/wwwcmt.cg) to construct species trees for the analysis of duplication loss events of *tRNA* genes. Species used for construction include *C. reinhardtii*, *H. laevis*, *G. pectorale*, *C. acuminata*, and *P. lenticularis* in the volvocales; *M. homosphaera*, *N. aquatica*, *B. giganteus*, *T. obliquus* and *A. judayi* in the sphaeropleales; *S. leibleinii* and *S. helveticum* in the chaetophorales; *O. cardiacum* in the oedogoniales and *F. terrestris* in the chaetopeltidales. After constructing the species tree, import it into Notung2.9 software, and reconcile and analyze the 20 subtype *tRNA* gene trees and species tree one by one to obtain gene duplication and deletion nodes.

## Results

### Analysis of the number and sequence length of *tRNA* in chloroplast genome

**Figure 2:**
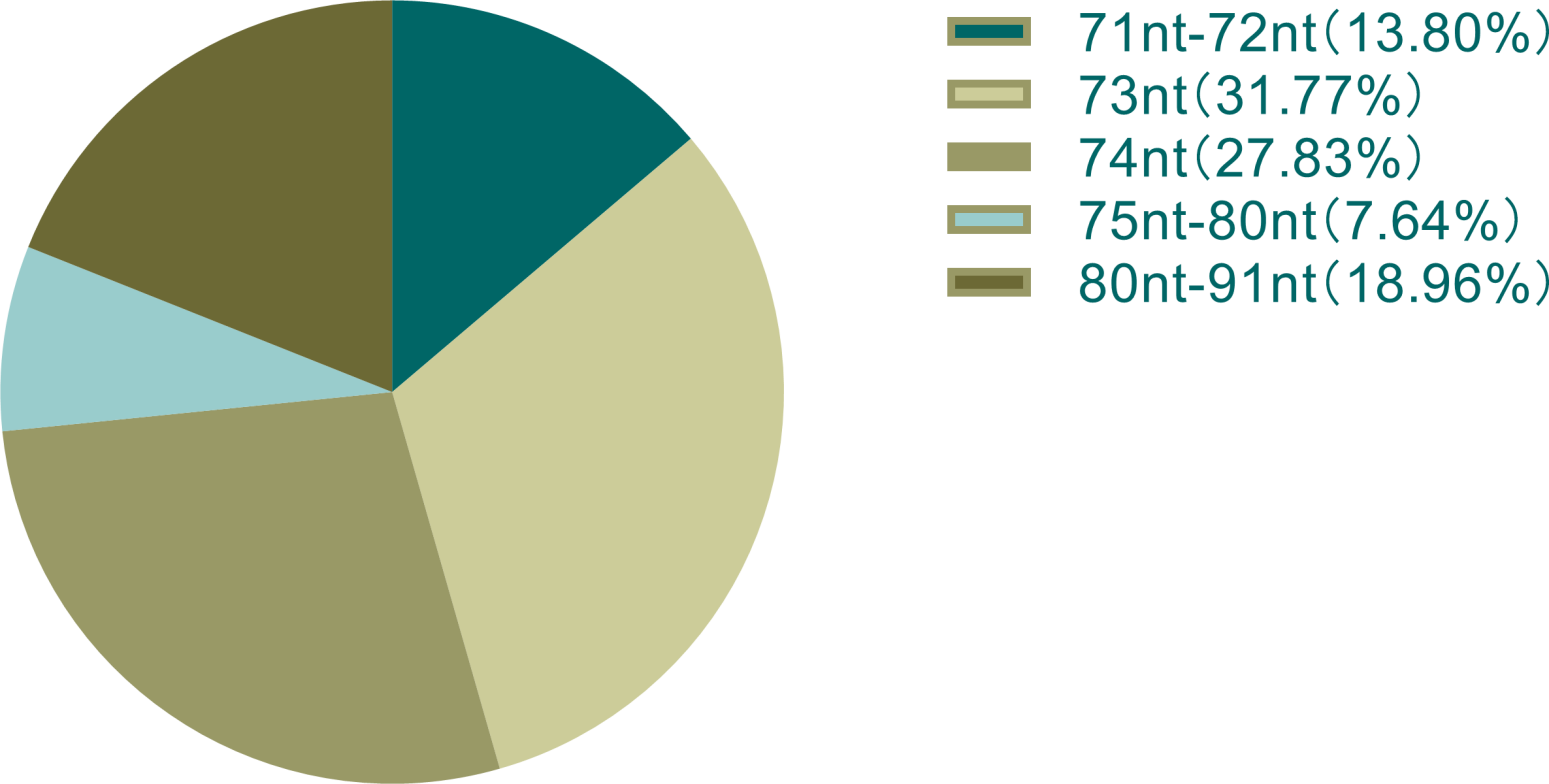
Distribution of the percentage of chloroplast *tRNA* gene sequence length in all species.

For a more comprehensive analysis of the evolution of chloroplast *tRNA* genomes in Chlorophyceae (the core Chlorophyceae), fourteen species from the NCBI Genome Database were randomly selected from the main five orders of Chlorophyceae. Genome Database of 14 species. Subsequently, the tRNAscan-SE software was used to perform detailed sequence analysis of the chloroplast genomes of 14 species. Through preliminary analysis, the results show that the chloroplast genomes of all species contain 28-32 *tRNAs*. *C. reinhardtii, C. acuminata, G. pectorale, N. aquatica, M. homosphaera* and *T. obliquus* encode 29 *tRNAs*; *A. judayi* and *O. cardiacum* encode 32 *tRNAs*; and *H. laevis, P. lenticularis, B. giganteus, S. leibleinii, S. helveticum* and *F. terrestris* encode 31, 30, 27, 30, 28 and 25 *tRNAs*, respectively (Table 1). For the predicted 406 chloroplast *tRNA* gene sequences (except for 3 sequences containing introns), the length of the chloroplast *tRNA* gene sequence of all species ranges from 71 nt (NC_005353.1). *tRNA* 29-CysGCA) to 91 nt, and maintains an average of 75 nt, of which 73 nt and 74 nt length contain 32% and 28% respectively. Not only that, among the 20 subtypes of *tRNAs*, *tRNA*^Glu^, *tRNA*^Lys^ and *tRNA*^V al^ contain 73 nt, 72 nt and 73 nt nucleotides, respectively. The nucleotides contained in *tRNA*^Asn^, *tRNA*^Asp^, *tRNA*^Cys^, *tRNA*^Gln^, *tRNA*^Gly^, *tRNA*^His^, *tRNA*^Phe^, *tRNA*^Pro^, *tRNA*^Thr^ and *tRNA*^Trp^ were all below the mean value (75 nt), while the nucleotides contained in *tRNA*^Leu^, *tRNA*^Ser^ and *tRNA*^Tyr^are all above 80 nt nucleotides (Fig. 2) (Data S1).

**Table 1:** Names of all species and the number of tRNAs in the chloroplast genome.

No.	Name of the order	Name of the family	Name of the genus	Name of the species	No. of *tRNAs*
1	Chlamydomonadales	Chlamydomonadaceae	Chlamydomonas	*C. reinhardtii*	29
2	Chlamydomonadales	Dunaliellaceae	Hafniomonas	*H. laevis*	31
3	Chlamydomonadales	Characiochloridaceae	Characiochloris	*C. acuminata*	29
4	Chlamydomonadales	Phacotaceae	Phacotus	*P. lenticularis*	30
5	Chlamydomonadales	Volvocaceae	Gonium	* G. pectorale*	29
6	Sphaeropleales	Neochloridaceae	Neochloris	*N. aquatica*	29
7	Sphaeropleales	Mychonastaceae	Mychonastes	*M. homosphaera*	29
8	Sphaeropleales	Sphaeropleaceae	Ankyra	*A. judayi*	32
9	Sphaeropleales	Bracteacoccaceae	Bracteacoccus	*B. giganteus*	27
10	Sphaeropleales	Scenedesmaceae	Tetradesmus	*T. obliquus*	29
11	Chaetophorales	Schizomeridaceae	Schizomeris	*S. leibleinii*	30
12	Chaetophorales	Chaetophoraceae	Stigeoclonium	*S. helveticum*	28
13	Oedogoniales	Oedogoniaceae	Oedogonium	*O. cardiacum*	32
14	Chaetopeltidales	Chaetopeltidaceae	Floydiella	*F. terrestris*	25

### Anticodon distribution of chloroplast *tRNA*

To determine the distribution of anticodons in chloroplast *tRNA*, we further analyzed the chloroplast genome sequences of 14 species in detail using tRNAscan-SE software. The results show that chloroplast *tRNAs* of all species contain 23-27 antisense codon types, while each species encodes 25-32 antisense codons. The most common types of anti-codons are: ACG (*tRNA*^Arg^), GAA (*tRNA*^Phe^), GTT (*tRNA*^Asn^), GTC (*tRNA*^Asp^), GTG (*tRNA*^His^), GAT (*tRNA*^Ile^), GTA (*tRNA*^Tyr^), GCA (*tRNA*^Cys^), CAT (*tRNA*^Met^), CCA (*tRNA*^Trp^), TGC (*tRNA*^Ala^), TCC (*tRNA*^Gly^), TGG (*tRNA*^Pro^), TGT (*tRNA*^Thr^), TAC (*tRNA*^V al^), TGA (*tRNA*^Ser^), TAG (*tRNA*^Leu^), TTT (*tRNA*^Lys^), TTG (*tRNA*^Gln^), GCT (*tRNA*^Ser^) and TCT (*tRNA*^Arg^). In addition, the results show that compared with the analyzed anticodons of other Chlorophyta chloroplast *tRNAs*, some anticodons are missing. Among them, GCC (*tRNA*^Gly^) is missing only in *N. aquatica*; TTC (*tRNA*^Glu^) is only missing in *F. terrestris*; while TAA (*tRNA*^Leu^) is missing in *B. giganteus, T. obliquus, F. terrestris, S. leibleinii* and *O. cardiacum* (Table 1).

### Multiple sequence alignment analysis of chloroplast *tRNA*

After categorizing the 20 subtypes of *tRNA*, the corresponding nucleotide sequence of each was analyzed by sequence alignment (Table 2). The results show that the Ψ-loop contains a conserved sequence of U-U-C-x-A in the secondary structure of the 20 *tRNA* isoforms. And the first position of most *tRNA* nucleotide sequence has G nucleotide, while *tRNA*^Asn^ and *tRNA*^Gln^ have U in the first position, *tRNA*^Trp^ and *tRNA*^V al^ have A in the first position, and *tRNA*^Pro^ has C in the first position. Subsequently, no identical nucleotide sequence was found at positions 2 to 7 of the acceptor arm. In the D-arm, 7 subtypes of *tRNA* were found to have a conserved sequence of GCNN (N represents any nucleotide), and there is a conserved sequence of AGU- (- represents any nucleotide or no nucleotide) in the D-loop. This is consistent with the reported results of the conserved consensus sequence ^7^GUGGCNNAGU^16^- starting with the 7th nucleotide of the acceptor arm in a typical *tRNA* (Laslett & Canback, 2004). In addition, it was found that *tRNA*^Ala^, *tRNA*^Arg^, *tRNA*^Asn^, *tRNA*^Gly^, *tRNA*^Lle^ and *tRNA*^phe^have the same nucleotide sequence as G-C-U-C in the D-arm.

**Table 2:** Sequence alignment of each secondary structure nucleotide of *tRNA*.

*tRNA* Isotypes	Acceptor Arm	D-arm	D-loop	AC- arm	Anti-codon loop	Variable loop	Ψ-arm	Ψ-loop
Ala	G-G-G-G-G-U-A	G-C-U-C	A-G-U-U-G-G-U-A	C-U-G-C-C	U-U-U-G-C-A-A	X-U-G-U-C	A-G-G-G-G	U-U-C-G-A-A-U
Arg	G-G-G-C-U-X-G	G-C-U-C	A-G-U-G-G-A-X-U-X_2_-A	X_3_-G-X	C-U-U-C-U-A-A	X-A-G-U-X	X_2_-G-G-G	U-U-C-G-A-A-U
Asn	U-C-C-U-C-A-G	G-C-U-C	A-G-U-G-G-U-A	G-U-C-G-G	C-U-G-U-U-A-A	U-G-G-U-C	G-U-A-G-G	U-U-C-A-A-G-U
Asp	G-G-G-A-U-U-G	G-U-U-C	A-A-U-U-G-G-U-U-A	C-C-G-C-C	C-U-G-U-C-A-C	A-A-G-U-U	G-C-G-G-G	U-U-C-G-A-G-U
Cys	G-G-C-G-G-C-A	G-C-C	A-A-G-U-G-G-U-A-A	G-A-G-G-A	U-U-G-C-A-A-A	U-A-U-U-C	C-C-C-A-G	U-U-C-G-A-A-U
Gln	U-G-G-G-G-C-G	G-C-C	A-A-G-U-G-G-U-A-A	G-U-G-G-U	U-U-U-U-G-G-U	C-A-U-U-C	G-X-A-G-G	U-U-C-G-A-A-U
Glu	G-C-C-C-C-C-A	G-U-C-U	A-G-A-G-G-C-C-U-A	C-C-U-C-C	C-U-U-U-C-A-C	G-A-A-A-C	G-G-G-G-A	U-U-C-G-A-A-U
Gly	G-C-G-G-A-U-X	G-C-U-C	A-G-U-X-G-G-U-A	C-X_2_-C-C	U-U-G-C-C-A-A	A-U-G-U-C	G-C-G-C-G	U-U-C-G-A-X-U
His	G-C-G-G-G-C-G	G-C-C	A-A-G-U-G-G-U-A-A	G-U-G-G-A	U-U-G-U-G-A-C	C-A-U-U-C	G-C-G-G-G	U-U-C-G-A-A-C
Leu	G-C-C-U-U-U-G	A-U-G	G-A-A-U-X-G-G-U-A-G-A	X_2_-G-G-X	U-U-U-A-A-A-A	U-G-C-U-X-U-X _3_-A-X-G-X-G-C-G-U	G-X-C-G-G	U-U-C-A-A-G-U
Lle	G-G-G-C-U-A-U	G-C-U-C	A-G-U-U-G-G-U-U-A	C-A-C-C-C	U-U-G-A-U-A-A	A-G-G-U-X-C	G-X-A-G-G	U-U-C-A-A-G-U
Lys	G-G-G-U-U-A-C	A-C-U-C	A-A-U-G-G-U-A	U-C-G-G	C-U-U-U-U-A-A	U-A-A-G-U-U	C-U-G-G-G	U-U-C-G-A-G-U
Met	X-G-C-A-G-X _2_	G-X_2_-C	A-G-U-C-G-G-X-U-A	U-X-C-G-X	C-U-C-A-U-A-A	A-U-G-U-C	G-C-A-G-G	U-U-C-A-A-X-U
phe	G-C-C-G-G-G-G	G-C-U-C	A-G-U-X-G-G-U-A	G-A-G-G-A	U-U-G-A-A-A-A	G-U-G-U-C	A-C-C-A-G	U-U-C-A-A-U-C
Pro	C-G-G-G-A-U-G	G-C-G-C	A-G-U-U-U-G-G-U-A	U-X-U-G-C	U-U-U-G-G-G-A	G-G-G-U-C	G-C-A-G-G	U-U-C-G-A-A-U
Ser	G-G-A-A-A-G-A	G-C-X	G-A-G-U-G-G-U-C-G-A-A	X-C-G-G-X	U-U-G-G-A-A-A	U-G-U-A-G-C-X _6_-G-X-U-X_4_-A-C-C	G-A-G-G-G	U-U-C-G-A-A-U
Thr	G-C-C-U-G-C-U	A-C-U-C	A-A-U-C-G-G-U-A	U-C-G-G-U	U-U-U-G-U-A-A	A-G-G-U-U	A-U-C-G-G	U-U-C-A-A-C-U
Trp	A-C-G-C-C-C-U	G-U-U-C	A-G-U-X-G-G-U-A	C-A-G-G-U	U-U-C-C-A-A	A-U-G-U-C	G-U-G-G-G	U-U-C-A-A-G-U
Tyr	G-G-G-U-C-G-A	C-C-C-G	A-G-U-X-G-G-U-U-A-A	G-C-G-G-A	U-U-G-U-A-A-A	U-G-G-C-U-X-A-X-G-C-C-U-A-C	G-C-U-G-G	U-U-C-G-A-A-U
Val	A-G-G-C-C-C-A	A-C-U-C	A-G-U-U-G-G-U-A	A-U-U-G-C	C-U-U-A-C-A-A	A-X-G-U-C	A-U-C-G-G	U-U-C-G-A-G-U

No conserved sequences were found in the anticodon arms and variable loops, but in the anticodon loops, a conserved U nucleotide was found at the second nucleotide. In addition to *tRNA*^Cys^, *tRNA*^Glu^, *tRNA*^Gly^ and *tRNA*^phe^, it was found that there are conserved nucleotide sequences of GG at positions 52 and 53 (that is, positions 4 and 5 of the Ψ-arm); for positions 54, 55 and 56, the 58th and 60th nucleotides (that is, the 1, 2, 3, 5, and 7 positions of the Ψ-arm), except for *tRNA*^His^ and *tRNA*^phe^, all have the conserved nucleotide sequence of U-U-C-x-A-x-U. Among them, the nucleotide sequence of *tRNA*^His^ at positions 54-60 is U-U-C-G-A-A-C; the nucleotide sequence of *tRNA*^phe^ at positions 54-60 is U-U-C-A-A-U-C.

### Analysis of the number of each structure of chloroplast *tRNA*

**Figure 3:**
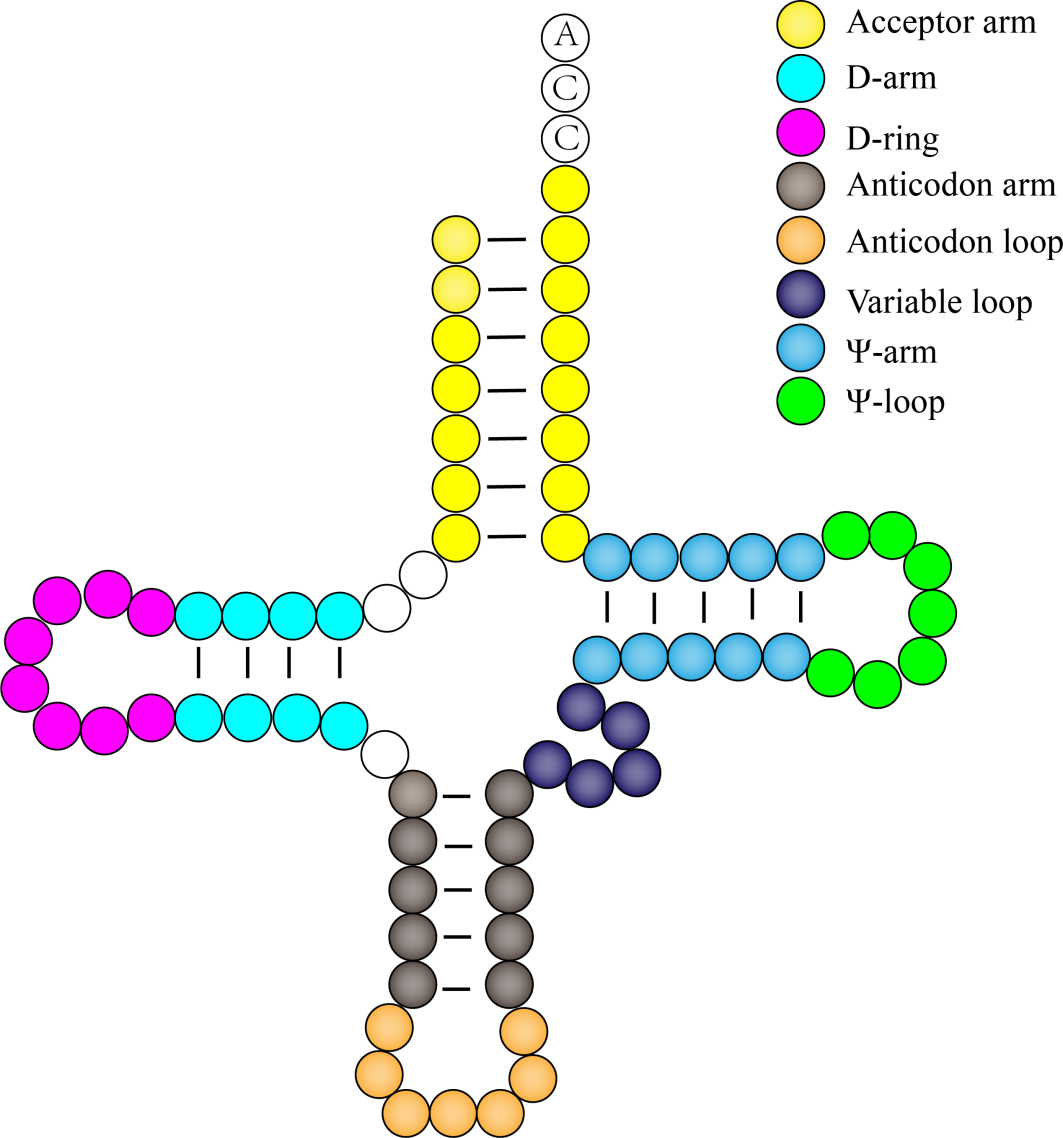
Nucleotide proportion diagram of each element of *tRNA* secondary structure. Among the 406 *tRNA* measured, the *tRNA* with seven nucleotide length in the receptor arm, anticodon loop and Ψ-loop accounted for 96.8%, 99.01% and 99.75% of the measured *tRNA*, respectively. The *tRNA* with four nucleotide length in D-arm accounted for 71.43%, and the *tRNA* with five nucleotide length in inverse curvature arm, variable loop and Ψ-arm accounted for 91.13%, 70.44% and 100%, respectively. The *tRNA* of D-loop nucleotide length of eight accounted for 24.63% of the detected *tRNA*.

After analyzing and sorting out the chloroplast *tRNAs* of 14 species, a total of 406 *tRNA* sequences (without 3 containing a group of introns) *tRNA* sequences were obtained. After statistical analysis of the number of nucleotides in each secondary structure of 406 *tRNAs*, we found that in the acceptor arm, the number of nucleotides is 6–7, of which 6-nucleotide *tRNA* accounts for 0.32%, 7-nucleotide *tRNA* accounted for 96.8%, which is consistent with the result that the number of acceptor arm nucleotides in the study is 7; In the D-arm, the number of nucleotides is 2–4, of which 3 nucleotides *tRNA* accounted for 28.3%, 4 nucleotides *tRNA* accounted for 71.4%, only NC_005353.1.*tRNA*^Cys^ D-arm is 2 nucleotides; In the D-ring, the number of nucleotides is 7–12, accounting for 12.8%, 24.6%, 44.6%, 7.1%, 11.6% and 0.25% respectively. Only NC_008372.1.*tRNA*^Tyr^ has 12 nucleotides. In the anticodon arm, the number of nucleotides is 4–5, of which *tRNA* containing 4 nucleotides accounted for 8.9%, and the proportion of *tRNA* containing 5 nucleotides was 91.1%. In the anticodon loop, the number of nucleotides is 7–9, 99.0% of the *tRNA* nucleotides are 7, and 0.73% of the *tRNA* nucleotides are 9. Similarly, only one *tRNA* has an anticodon ring structure with a nucleotide number of 8. For the variable loop, as the structure with the largest number of nucleotide changes in the *tRNA* secondary structure, among the 406 *tRNAs* tested, the number of nucleotides in the variable loop ranged from 3–22 nt, but 77.44% *tRNA*, the number of nucleotides in the variable loop is 5. In addition, we enumerate the secondary structures of three different *tRNAs* with variable loops/stems (Fig. S1). In the Ψ-arm, only one *tRNA* has 4 nucleotides, and the remaining 405 *tRNAs* have 5 nucleotides; in the Ψ-ring, the number of *tRNA* nucleotides is all 7, and there is no special structure of the variation. Therefore, it is speculated that the Ψ-loop in the *tRNA* secondary structure is the most conserved structural unit, which is consistent with the results of the previous multiple sequence alignments (Fig. 3).

### Analysis of introns in chloroplast *tRNA*

**Figure 4:**
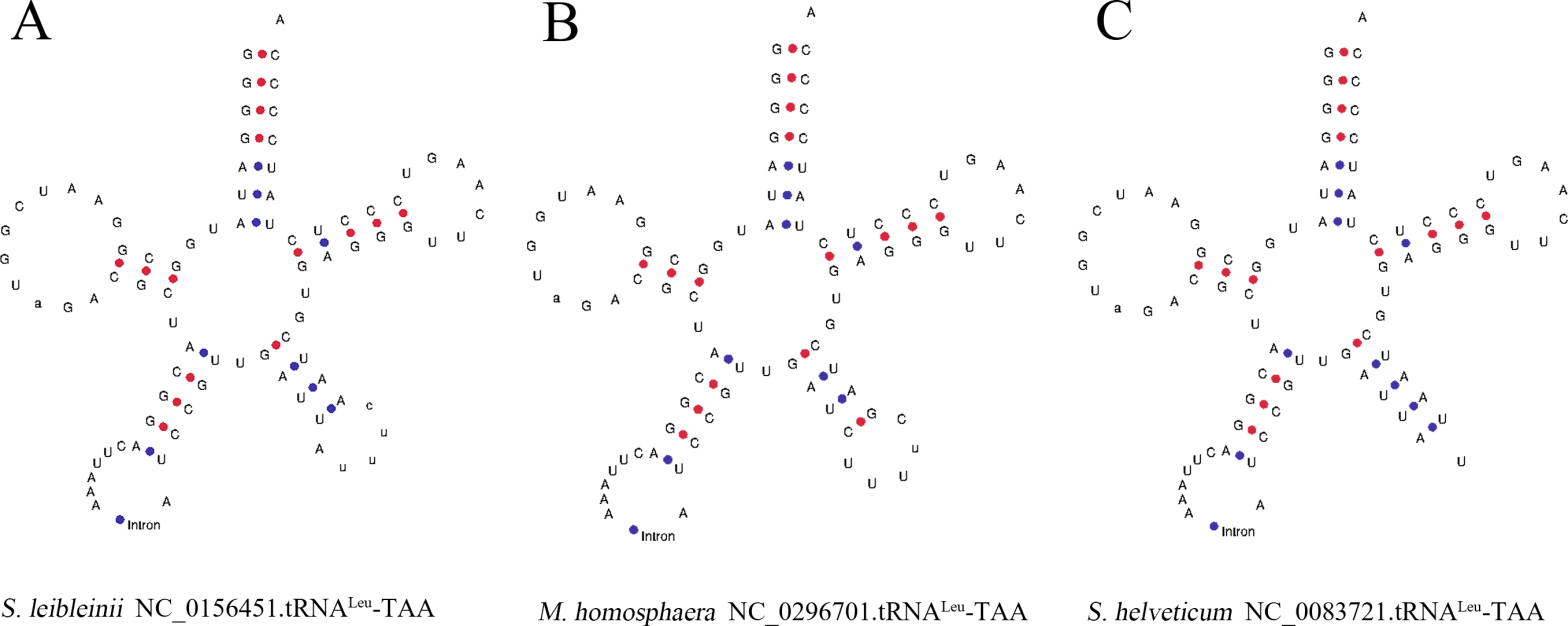
Diagram of *tRNA* secondary structure containing introns. Among the chloroplast *tRNA* genomic sequences analysed from green algae, there are three sets of *tRNA* sequences with introns whose positions are all located in the anticodon loop. (A)–(C) show the secondary structure of these three *tRNA* sequences, with red and blue markers indicating the G–C and A–U bonds, respectively. (A) represents *M. homosphaera*, NC_029670.1.*tRNA*^Leu^-TAA. (B) represents *S. helveticum*, NC_008372.1. *tRNA*^Leu^-TAA. (C) represents *S. leibleinii*, NC_015645.1 *tRNA*^Leu^- TAA.

**Figure 5:**
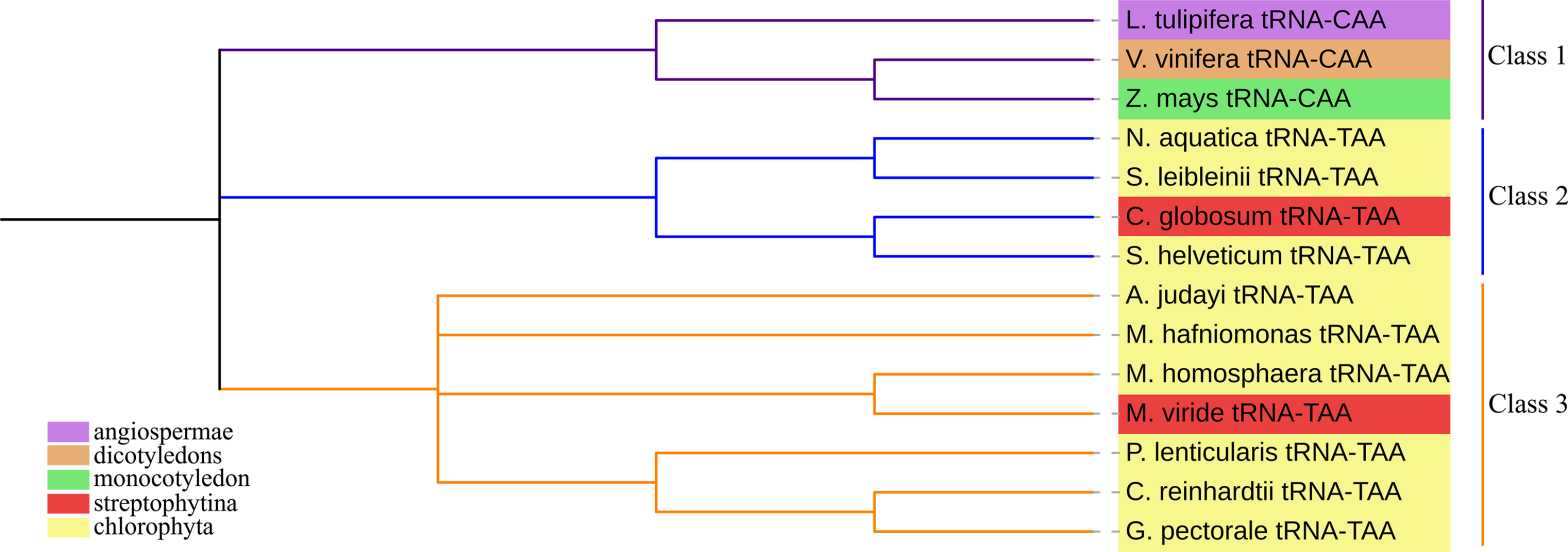
The phylogenetic tree of *tRNA*^Leu^. The phylogenetic tree of *tRNA*^Leu^ is divided into three classes. From the tree, it can be seen that the *tRNA*^Leu^ containing introns is the closest in relation to each other. The classes represented by the species in the tree are colored in the lower left illustration.

After analyzing the chloroplast *tRNA* genome sequence of all Chlorophyta, we found that the chloroplast *tRNA* of *M. homosphaera* (NC_029670.1) .*tRNA*^Leu^ (TAA)) in the sphaeropleales, *S. leibleinii* (NC_015645.1). *tRNA* Leu (TAA)) and *S. helveticum* (NC_008372.1). *tRNA*^Leu^ (TAA)) in the chaetophorales contain introns (Fig. 4). These three groups of introns are all located in the anticodon loop of *tRNA*, with nucleotide lengths of 174, 253, and 243, respectively. The starting points in the *tRNA* structure are 38, 39, 39, and from the analysis results, the scores (Internal scores) of these three *tRNAs* are 35.9 bits, 20.7 bits and 33.4 bits, respectively, indicating that they may not be functional. Subsequently, we selected the chloroplast genome sequences of five plants from the NCBI genome database, streptophytina *Chaetosphaeridium globosum* (NC_004115), *Mesostigma viride* (NC_002186), angiospermae *Liriodendron tulipifera* (NC_008326), monocotyledon *Zea mays* (NC_001666), and dicotyledons *Vitis vinifera* (NC_007957.1). The chloroplast *tRNA* gene sequences of these 5 plants were predicted by tRNAscan-SE software. It was found that *tRNA*^Leu^ (TAA), which also contains introns, was found in streptophytina, but this *tRNA* was not found in the chloroplast genome sequence of angiospermae *L. tulipifera*, monocotyledon *Z. mays*, and dicotyledons *A. thaliana*. Therefore, it is speculated that *tRNA*^Leu^ (TAA) containing introns in the chloroplast genome of green plants first appeared in Chlorophyta and Streptophytina. In addition, the chloroplast genome *tRNA*^Leu^ of angiosperms, monocots, and dicots was used as a peripheral group to construct a phylogenetic tree of *tRNA*^Leu^ (Fig. 5). It is clear from tree that the *tRNA*^Leu^ containing introns are close to each other on the evolutionary branch.

### Transition, transversion bias analysis

To better understand *tRNA* evolution, we performed MCL conversion and escape bias analyses on 20 *tRNA* isoforms measured in chloroplasts and compared the results with those of *tRNA* analysis in cyanobacteria and monocot chloroplasts (Mohanta et al., 2017; Mohanta et al., 2019). The results showed that although green algae and monocotyledon belong to eukaryotes, the transition and transversion results were consistent with the analysis results of cyanobacteria *tRNA*, and the chloroplast *tRNA* conversion rate of green algae was higher than the transversion rate. On the contrary, it is inconsistent with the chloroplast *tRNA* analysis results of monocotyledonous plants, because we found that in the chloroplast *tRNA* analysis results of monocotyledonous plants, the transformation rate and transversion rate of *tRNA*^Ala^, *tRNA*^Asn^, *tRNA*^Asp^, *tRNA*^His^, *tRNA*^Phe^ and *tRNA*^Pro^ were almost equal, but did not appear in the chloroplast *tRNA* analysis results of green algae. In addition, from the data analysis, we found that the transition rate of *tRNA*^Asp^ and *tRNA*^V al^ is significantly higher than the transition rate. The transition rate between A ↔G in *tRNA*^V al^ is as high as 30.95%, second only to the transition rate of C ↔U (33.32%) in *tRNA*^Trp^. Not only that, the transition rate between A ↔C in *tRNA*^V al^ is the lowest among all *tRNAs* analyzed, only 0.58% (Table 3).

### Phylogenetic tree analysis of Chlorophyta chloroplast *tRNA*

**Figure 6:**
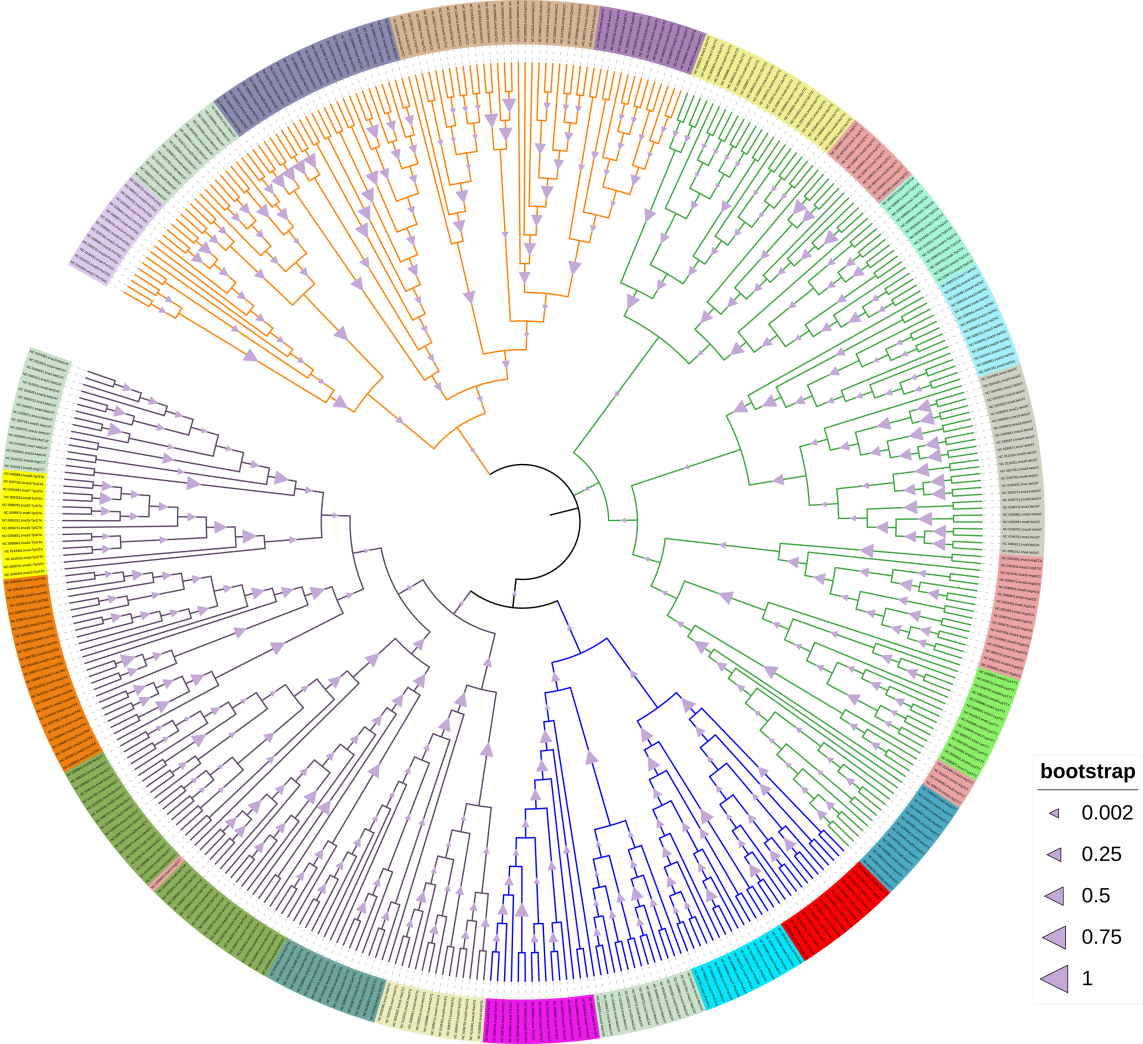
Green algae chloroplast *tRNA* phylogeny tree. In the constructed phylogenetic tree, all *tRNAs* are divided into four clusters according to the evolutionary relationship of the phylogenetic tree. It is speculated from the branching display of the phylogenetic tree that the fourth cluster of *tRNA* is relatively advanced in the evolutionary relationship. There are mutations in the evolution of green algae chloroplast *tRNA*, with *tRNA*^Met^, *tRNA*^Arg^ and *tRNA*^Ser^ being the most obvious.

**Figure 7:**
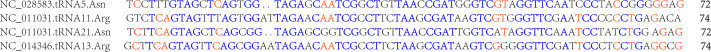
Sequence alignment of *tRNA*^Arg^ (TCT) and *tRNA*^Asn^ (GTT). The homologous sequence of *tRNA*^Arg^ (TCT) and *tRNA*^Asn^ (GTT) is up to 80%. The species were *H. laevis* (NC_028583.1), *O. cardiacum* (NC_011031.1), *F. terrestris* (NC_014342.1).

**Figure 8:**
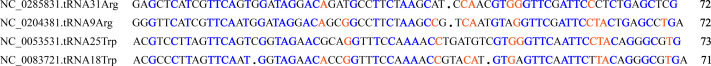
Sequence alignment of *tRNA*^Arg^ (TCT) and *tRNA*^Trp^ (CCA). The homologous sequence of *tRNA*^Arg^ (TCT) and *tRNA*^Trp^ (CCA) was 77.36%. The species were: *H. laevis* (NC_028583.1), *G. pectoralis* (NC_020438.1), *C. reinhardtii* (NC_005353.1), *S. helveticum* (NC_008372.1).

After aligning and analyzing 406 *tRNA* sequences, a phylogenetic tree was constructed using all *tRNA* genome sequences. After sorting and analyzing, according to the evolutionary relationship of the phylogenetic tree, all *tRNAs* are divided into four clusters, the first cluster of *tRNAs* are: *tRNA*^Met^, *tRNA*^Arg^, *tRNA*^Tyr^, *tRNA*^Leu^, *tRNA*^Ser^; the second cluster of *tRNAs* are: *tRNA*^His^, *tRNA*^Met^, *tRNA*^Gln^, *tRNA*^Cys^ , *tRNA*^Phe^, *tRNA*^Thr^; the third cluster of *tRNAs* are: *tRNA*^Arg^, *tRNA*^His^, *tRNA*^Gln^, *tRNA*^Glu^, *tRNA*^Trp^, *tRNA*^V al^, *tRNA*^lle^, *tRNA*^Asn^, *tRNA*^Lys^; the fourth cluster of *tRNAs* are: *tRNA*^Ala^, *tRNA*^Asp^, *tRNA*^Gly^, *tRNA*^Pro^, *tRNA*^Met^ (Fig. 6). In addition, we can find several interesting phenomena from the evolutionary tree: firstly, there are multiple branches of *tRNA*^Arg^ and *tRNA*^Met^ in the entire phylogenetic tree. It is speculated that this may be due to multiple mutations of these two *tRNAs* during the evolution process. Secondly, in the third cluster, *tRNA*^Arg^ (TCT) is the closest to *tRNA*^Asn^ (GTT) and *tRNA*^Trp^ (CCA). From the sequence comparison results, the sequence similarity between *tRNA*^*Arg*^ (TCT) and *tRNA* ^*Asn*^ (GTT) was very high, reaching 80%. Similarly, the sequence similarity between *tRNA*^*Arg*^ (TCT) and *tRNA*^*Trp*^ (CCA) was also very high, reaching 77.36% (Figs. 7 and 8). The evolutionary relationship between *tRNA*^Arg^(TCT) and *tRNA*^Arg^(ACG) (TCG) is closer than that of other *tRNA*^Arg^ in the evolutionary tree, so we speculate that *tRNA*^Arg^ (TCT) is evolved from *tRNA*^Arg^ (ACG) (TCG); *tRNA*^Arg^ (CCT) has two points branches, one of which is similar to *tRNA*^Met^ in genetic relationship, and the other branch is likely to be present in the branches of *tRNA*^Ser^ due to mutations during evolution. In addition, in the evolutionary tree, the branches of *tRNA*^Ser^ are obviously denser, which indicates that *tRNA*^Ser^ seems to be susceptible to mutations. The secondary structure prediction results of *tRNA*^Ser^ by tRNAscan-SE software also support it. There are three branches of *tRNA*^Met^ (CAT), which are closely related to *tRNA*^Arg^ (CCT), *tRNA*^Thr^ (TGT) and *tRNA*^Pro^ (TGG).

**Table 3:** Transition rate/transversion rate of *tRNA*.

Ala	A	U	C	G	Leu	A	U	C	G
A	___	*5.04*	*4.52*	21.42	A	___	*3.93*	*3.65*	18.60
U	*3.77*	___	12.51	*6.08*	U	*3.88*	___	16.34	*4.88*
C	*3.70*	13.95	___	*6.08*	C	*3.88*	17.60	___	*4.88*
G	13.29	*5.04*	*4.52*	___	G	14.79	*3.93*	*3.65*	___
Arg					Lys				
A	___	*4.98*	*4.96*	17.89	A	___	*4.10*	*3.19*	19.54
U	*4.50*	___	13.85	*5.78*	U	*3.67*	___	13.68	*3.54*
C	*4.50*	13.91	___	*5.78*	C	*3.67*	17.56	___	*3.54*
G	13.93	*4.98*	*4.96*	___	G	20.25	*4.10*	*3.19*	___
Asn					Met				
A	___	*2.96*	*2.44*	20.14	A	___	*7.42*	*7.39*	10.19
U	*2.25*	___	20.19	*3.57*	U	*6.89*	___	10.70	*8.17*
C	*2.25*	24.50	___	*3.57*	C	*6.89*	10.74	___	*8.17*
G	12.70	*2.96*	*2.44*	___	G	8.60	*7.42*	*7.39*	___
Asp					Phe				
A	___	*1.32*	*1.39*	28.74	A	___	*3.88*	*3.61*	17.25
U	*0.96*	___	22.97	*1.76*	U	*3.64*	___	18.26	*4.87*
C	*0.96*	21.68	___	*1.76*	C	*3.64*	19.62	___	*4.87*
G	15.74	*1.32*	*1.39*	___	G	12.89	*3.88*	*3.61*	___
Cys					Pro				
A	___	*2.60*	*2.71*	13.68	A	___	*5.18*	*4.39*	21.70
U	*2.50*	___	27.16	*2.97*	U	*3.53*	___	12.50	*6.73*
C	*2.50*	26.08	___	*2.97*	C	*3.53*	14.74	___	*6.73*
G	11.51	*2.60*	*2.71*	___	G	11.38	*5.18*	*4.39*	___
Gln					Ser				
A	___	*5.12*	*5.62*	16.35	A	___	*6.81*	*5.59*	12,38
U	*3.09*	___	18.44	*6.49*	U	*5.11*	___	13.16	*7.48*
C	*3.09*	16.78	___	*6.49*	C	*5.11*	16.03	___	*7.48*
G	7.80	*5.12*	*5.62*	___	G	8.47	*6.81*	*5.59*	___
Glu					Thr				
A	___	*2.98*	*4.64*	20.10	A	___	*4.96*	*3.39*	15.37
U	*3.17*	___	21.18	*4.21*	U	*4.22*	___	14.38	*4.09*
C	*3.17*	13.59	___	*4.21*	C	*4.22*	21.06	___	*4.09*
G	15.11	*2.98*	*4.64*	___	G	15.87	*4.96*	*3.39*	___
Gly					Trp				
A	___	*3.85*	*3.97*	21.20	A	___	*2.95*	*2.43*	9.83
U	*2.81*	___	17.96	*4.57*	U	*2.40*	___	27.45	*3.03*
C	*2.81*	17.40	___	*4.57*	C	*2.40*	33.32	___	*3.03*
G	13.04	*3.85*	*3.97*	___	G	7.78	*2.95*	*2.43*	___
His					Tyr				
A	___	*2.66*	*3.36*	25.29	A	___	*2.95*	*2.86*	23.69
U	*2.05*	___	20.89	*3.92*	U	*2.27*	___	18.95	*3.84*
C	*2.05*	16.57	___	*3.92*	C	*2.27*	19.50	___	*3.84*
G	13.25	*2.66*	*3.36*	___	G	14.00	*2.95*	*2.86*	___
lle					Val				
A	___	*4.46*	*3.77*	15.65	A	___	*0.67*	*0.58*	30.95
U	*4.26*	___	16.49	*4.93*	U	*0.61*	___	17.80	*0.73*
C	*4.26*	19.49	___	*4.93*	C	*0.61*	20.36	___	*0.73*
G	13.51	*4.46*	*3.77*	___	G	25.70	*0.67*	*0.58*	___

### Analysis of duplication and loss of chloroplast *tRNA*

In the evolution of *tRNA*, there are not only transformation and transversion events, but also duplication and loss events. Therefore, studying the duplication and loss of chloroplast *tRNA* is very important for its evolution. After analysis, we’ve got the loss and duplication tree (Fig. S2). And the results showed that among all the *tRNAs* tested, duplications events occurred a total of 151 times, conditional duplications events occurred a total of 101 times, and losses events occurred a total of 311 times (Table 4) (Data S2). Therefore, we speculate that a large number of genes may be lost during the evolution of the chloroplast *tRNA*. In addition, we found that *tRNA*^Met^ had a higher D/L (duplication/loss) score than other *tRNAs*, while *tRNA*^Pro^ and *tRNA*^Try^ had a lower D/L score, which may be due to the fact that *tRNA*^Pro^ and *tRNA*^Try^ are more conserved during evolution.

**Table 4:** Duplication and loss events of each subtype *tRNA*.

*tRNA*	D/L score	Duplications	Conditional duplications	Losses
Ala	39.0	16	3	15
Arg	31.5	9	10	18
Asn	19.0	4	4	13
Asp	20.0	4	3	14
Cys	18.0	4	2	12
Glu	43.0	14	3	22
Gln	26.5	7	3	16
Gly	50.5	13	7	31
His	27.0	6	3	18
Ile	22.0	12	6	4
Leu	29.0	8	9	17
Lys	13.5	3	3	9
Met	55.5	15	12	33
Phe	17.0	4	2	11
Pro	9.0	2	7	6
Ser	43.5	13	9	24
Thr	19.0	4	3	13
Trp	10.0	2	5	7
Tyr	24.0	6	2	15
Val	20.5	5	5	13

## Discussion

No *tRNAs* of the two subtypes selenocysteine and possible suppressor were found to be present in the analyzed chloroplast genomes of the Chlorophyceae. There are 11 subtypes of *tRNAs* that encoded only one *tRNA* in 14 species, and 9 subtypes of *tRNAs* encoded different numbers of *tRNAs*. Among these 9 subtypes of *tRNAs*, the *tRNA*^Glu^ was not found in *F. terrestris*. It is speculated that in *F. terrestris*, the codon recognition of glutamate may rely on the introduction of *tRNA* from the cytoplasm. To prove that the chloroplast is the evidence that *tRNA* can be introduced from the cytoplasm (Wolfe, Morden & Palmer, 1992; Smith, 2009). *tRNA*^Arg^ appeared 5 times in *O. cardiacum*, *tRNA*^Ser^ appeared 4 times in *S. leibleinii*, and *tRNA*^Met^ appeared 4 times in *P. lenticularis*. Therefore, based on the fact that *tRNA*^Arg^, *tRNA*^Ser^ and *tRNA*^Met^ occur frequently in the species, we speculate that it may be because these three groups of *tRNA* are more prone to mutation during evolution, which is consistent with the results of the constructed phylogenetic tree. The characteristic of *tRNA* is a clover secondary structure composed of three hairpin loops and a terminal spiral stem, and the length of *tRNA* is generally between 75–95 nt (Goodenbour & Pan, 2006). In our study, it was found that 73.4% of the sequence lengths of *tRNA* of the 14 chloroplast species were less than 75 nt, which may have a great relationship with the *tRNA* has been in a conserved evolutionary stage.

Anticodons are of great significance in the process of protein synthesis. Anticodons and codons can introduce specific amino acids into the ribosome through base pairing (Sciarrino & Sorba, 2012). At the same time, anticodons and codons also differ greatly in number and evolutionary conservation. Anticodons also have variability in different organisms and organelles (Tong & Wong, 2004). In the 14 species analyzed, the anticodons were missing to varying degrees. At the same time, it was discovered that the missing *tRNA*^Gly^ (GCC) anticodon in *N. aquatica* was compensated by the *tRNA*^Gly^ (TCC) anticodon. The missing anticodon *tRNA*^Leu^ (TAA) in *B. giganteus*, *T. obliquus*, *F. terrestris*, *S. leibleinii* and *O. cardiacum* is compensated by the anticodon *tRNA*^Leu^ (TAG). But only in *F. terrestris* neither the anticodon *tRNA*^Glu^ (TTC) nor the anticodon *tRNA*^Glu^ (CTC) was found. In the chloroplast genome of Chlorophyceae, the gene sequences of *tRNA*^Asn^, *tRNA*^Asp^, *tRNA*^Cys^, *tRNA*^Glu^, *tRNA*^His^, *tRNA*^Lys^ and *tRNA*^Thr^ of the 20 subtypes of *tRNAs* of 14 species maintain relative conservation. In addition, it was also found that the variable loops of the three *tRNAs* were significantly longer than those of other *tRNAs*, which may be due to the fact that the variable loops have a certain influence on the orientation of the L-shaped tertiary structure of *tRNAs* (Bessho et al., 2007). Among them, the variable loop of *tRNA*^Ser^ is the longest and is not conserved, which results in that *tRNA*^Ser^ is susceptible to mutation in the phylogenetic tree.

As an important feature to distinguish prokaryotic genes, introns are widely present in eukaryotic genes, and a small amount are present in prokaryotes (Dai et al., 2003). The existence of introns in genes and their conservation information are closely related to the evolutionary origin and evolutionary relationship of species genes. For a long time, the main functions and research directions of introns have focused on the direction of gene expression regulation and selective splicing mechanism (Toor et al., 2008; Li et al., 2015; Swagata et al., 2018), but studies on introns as indicators of species evolution are relatively rare. The size and number of introns are not fixed in eukaryotic genes (Wu et al., 2013). The different ways in which repetitive sequences accumulate in plants and animals give rise to the fact that the length and number of introns in plants is much smaller than in higher animals (Wendel et al., 2002). Intron-containing *tRNA* genes are found in all kingdoms of life (Schmidt & Matera, 2020), and are rare in higher plants, but in Chlorophyta, more than half of *tRNA* genes contain an intron (Michaud et al., 2011). The distribution of introns in *tRNA* genes is also diverse, and can be located in different positions of *tRNA* genes, while the tRNAscan-SE server is difficult to accurately predict *tRNA* genes whose introns are not 37/38 and split *tRNA* genes (Sugahara et al., 2006). Therefore, in the process of analyzing the *tRNA* of the chloroplast genome of Chlorophyta, we only found that part of the *tRNA* genome contains a set of introns in the anti-codon ring. At the same time, based on the analysis scores of the tRNAscan-SE server, it is found that the scores of these three groups of *tRNA* are relatively low. It is speculated that these three groups of introns are not functional.

In the constructed *tRNA* phylogenetic tree, we found that the chloroplast *tRNA* of Chlorophyta was consistent with the *tRNA* of monocotyledonous and gymnospermatic chloroplasts in evolutionary pattern, with a common multi-phylogenetic pattern, and was rooted in a multiple common ancestor (Fig. 6). However, in the phylogenetic map of chloroplast genome constructed by us (Fig. S3), it was found that the branches of other species were not consistent with the branches of species tree except *S. Leibleinii* and *S. helveticum* in Chaetophorales, which indicated that the chloroplast evolution of chlorophyta was not inconsistent with the evolution pattern and direction of species evolution.

The conversion between nucleotides (U ↔C, A ↔G) and transversion (U ↔A, U ↔G, C ↔A, C ↔G) are important indicators for analyzing the evolution of *tRNA* genes. Research usually uses MAGE X software analysis and statistics. The MAGE X analysis in the data shows that the frequency of the nuclear genome of drosophilid is twice as high as the frequency of transversion (Begun et al., 2007). In the *DNA* sequences of many genomes, cyanobacteria *tRNA* and some monocot chloroplast *tRNA* genes, transition occurs more frequently than transversion occurs (Gojobori, Li & Dan, 1982; Wakeley, 1994; Mohanta et al., 2017; Mohanta et al., 2019). In this study, the MAGE X software measured the rate of transition/transversion greater than k1 (Purines) and K2 (Pyrimidines), indicating a higher frequency of transition than transversion in the chloroplast *tRNA* gene of Chlorophyta, which is similar to the results of the above analysis (Table 5). In the process of species evolution, gene duplication and loss affect the change of species genes. Gene duplication is an effective way to generate new gene functions in the genome, and gene loss can effectively shape gene families (Rasmussen & Kellis, 2012; Teufel, Liu & Liberles, 2016). In the event of gene sequence duplication loss in Chlorophyta chloroplast *tRNA*, *tRNA*^Met^, *tRNA*^Arg^ and *tRNA*^Ser^ all occurred at a high level, which may lead to multiple appearances of these three *tRNAs* in the chloroplast genome and active evolution in the phylogenetic trees constructed.

**Table 5:** The rate ratio of transition/transversion k1 (purines) and k2 (pyrimidines).

*tRNA*	k1 (Purines)	k2 (Pyrimidines)
Ala	3.526	2.766
Arg	3.098	2.794
Asn	5.635	8.266
Asp	16.319	16.478
Cys	4.611	10.021
Gln	2.521	3.279
Glu	4.769	4.56
Gly	4.638	4.522
His	6.45	6.219
Leu	3.815	4.48
Lle	3.174	4.368
Lys	5.526	4.286
Met	1.248	1.447
phe	3.541	5.06
Pro	3.223	2.845
Ser	1.656	2.355
Thr	3.76	4.243
Trp	3.242	11.299
Tyr	6.167	6.616
Val	42.19	30.49

## Conclusions

Through the sequence variation and evolutionary analysis of the chloroplast genome *tRNA* of chlorophyceae, we found that the chloroplast genome contains 28-32 *tRNAs*, and the length of the gene sequence ranges from 71 nt to 91 nt. There are 23–27 anticodon types of *tRNAs*, and some *tRNAs* have missing anticodons that are compensated for by other types of anticodons of that *tRNA*. In addition, three *tRNAs* were found to contain introns in the anticodon loop of the *tRNA*, but scored poorly when analyzed by the tRNAscan-SE software. Therefore, we speculate that these introns are not functional. After multiple sequence alignment, the Ψ-loop is the most conserved structural unit in the *tRNA* secondary structure, containing mostly U-U-C-x-A-x-U conserved sequences. The number of transitions in *tRNA* is higher than the number of transversions. In the gene duplication and gene loss analysis, it was found that green algal chloroplast *tRNAs* may have undergone substantial gene loss during the course of evolution. According to the constructed phylogenetic tree, it was found that there were mutations in the evolution of chlorophyta chloroplast *tRNA*, with *tRNA*^Met^, *tRNA*^Arg^ and *tRNA*^Ser^ being the most obvious.

##  Supplemental Information

10.7717/peerj.11524/supp-1 Supplemental Information 1 tRNA secondary structures of different variable loops/arms In the 409 groups of *tRNA* secondary structures we analyzed, in addition to the *tRNA* sequences with introns, we also surprisingly found that there were some *tRNA* secondary structures with abnormal structures. Fig. A–C is the secondary structure diagram of the three *tRNA* sequences of *C. cuminata* (tRNA ^Leu^-TAG, tRNA ^Arg^-ACG, tRNA ^Ser^-GCT), and their variable loops/arms are different. The red and blue markers indicate the G–C and A–U bonds, respectively.

10.7717/peerj.11524/supp-2 Supplemental Information 2 The loss and duplication tree 252 duplication events (duplication and conditional duplication) are detected in all of the gymnosperm chloroplast *tRNA* genes, and gene loss events are detected with 311. Blue, Duplication events; Gray, Loss events; D, Duplication node; cD, Conditional Duplication node.

10.7717/peerj.11524/supp-3 Supplemental Information 3 The phylogenetic map of chloroplast genome In the phylogenetic map of chloroplast genome, it was found that the branches of other species were not consistent with the branches of species tree except *S. Leibleinii* and *S. helveticum* in Chaetophorales, which indicated that the chloroplast evolution of chlorophyta was not inconsistent with the evolution pattern and direction of species evolution.

10.7717/peerj.11524/supp-4 Supplemental Information 4 Raw data for Fig. 2 The tRNA type of the species as well as the length of the tRNA sequence.

10.7717/peerj.11524/supp-5 Supplemental Information 5 Raw data for Table 4 The specific occurrence of replication events, conditional replication events and loss events for subtypes of tRNA for all species

10.7717/peerj.11524/supp-6 Supplemental Information 6 Anticodon distribution of chloroplast *tRNA*

## Additional Information and Declarations

### Data Availability

The following information was supplied regarding data availability: The raw measurements are available in the Supplemental Files.
