# Supplementary figures and images for: Structural variation and evolution of chloroplast tRNAs in green algae
Source: PeerJ. 2021 Jun 1;9:e11524. doi: 10.7717/peerj.11524 (PMC8176911; doi:10.7717/peerj.11524)

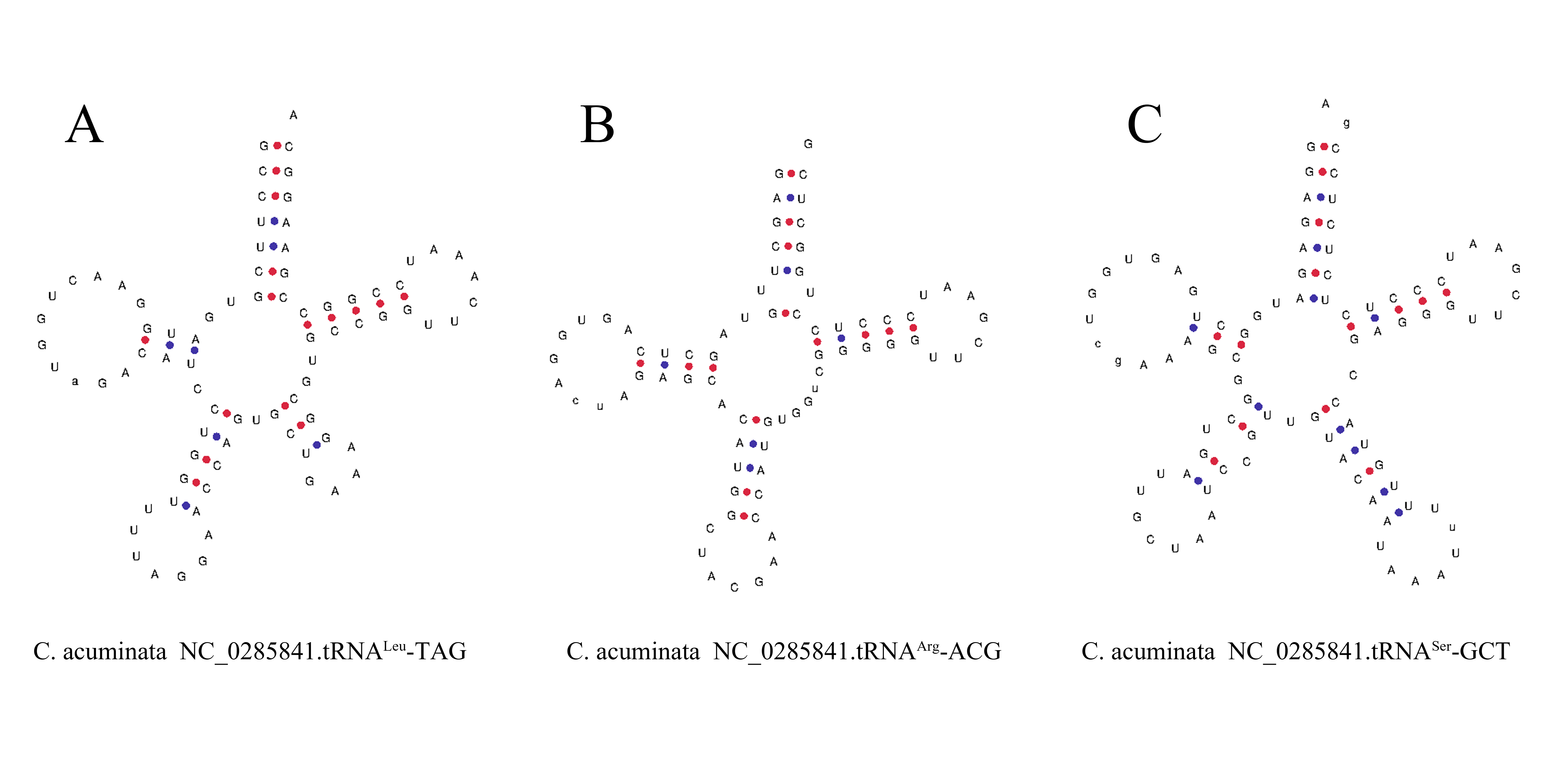

Supplement: Supplemental Information 1 — In the 409 groups of tRNA secondary structures we analyzed, in addition to the tRNA sequences with introns, we also surprisingly found that there were some tRNA secondary structures with abnormal structures. Fig. A–C is the secondary structure diagram of the three tRNA sequences of C. cuminata (tRNA Leu-TAG, tRNA Arg-ACG, tRNA Ser-GCT), and their variable loops/arms are different. The red and blue markers indicate the G–C and A–U bonds, respectively. [file peerj-09-11524-s001.png]

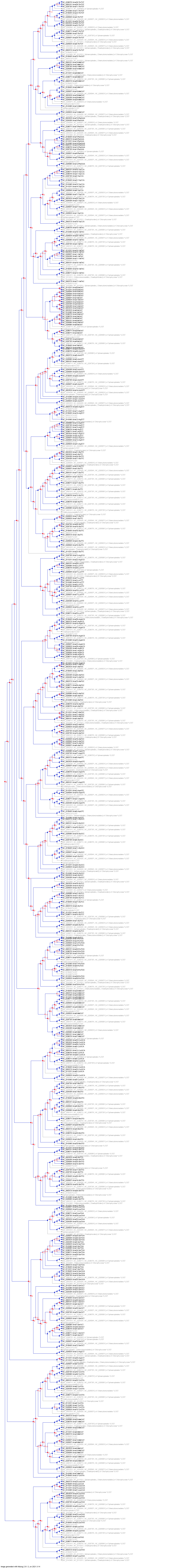

Supplement: Supplemental Information 2 — 252 duplication events (duplication and conditional duplication) are detected in all of the gymnosperm chloroplast tRNA genes, and gene loss events are detected with 311. Blue, Duplication events; Gray, Loss events; D, Duplication node; cD, Conditional Duplication node. [file peerj-09-11524-s002.png]

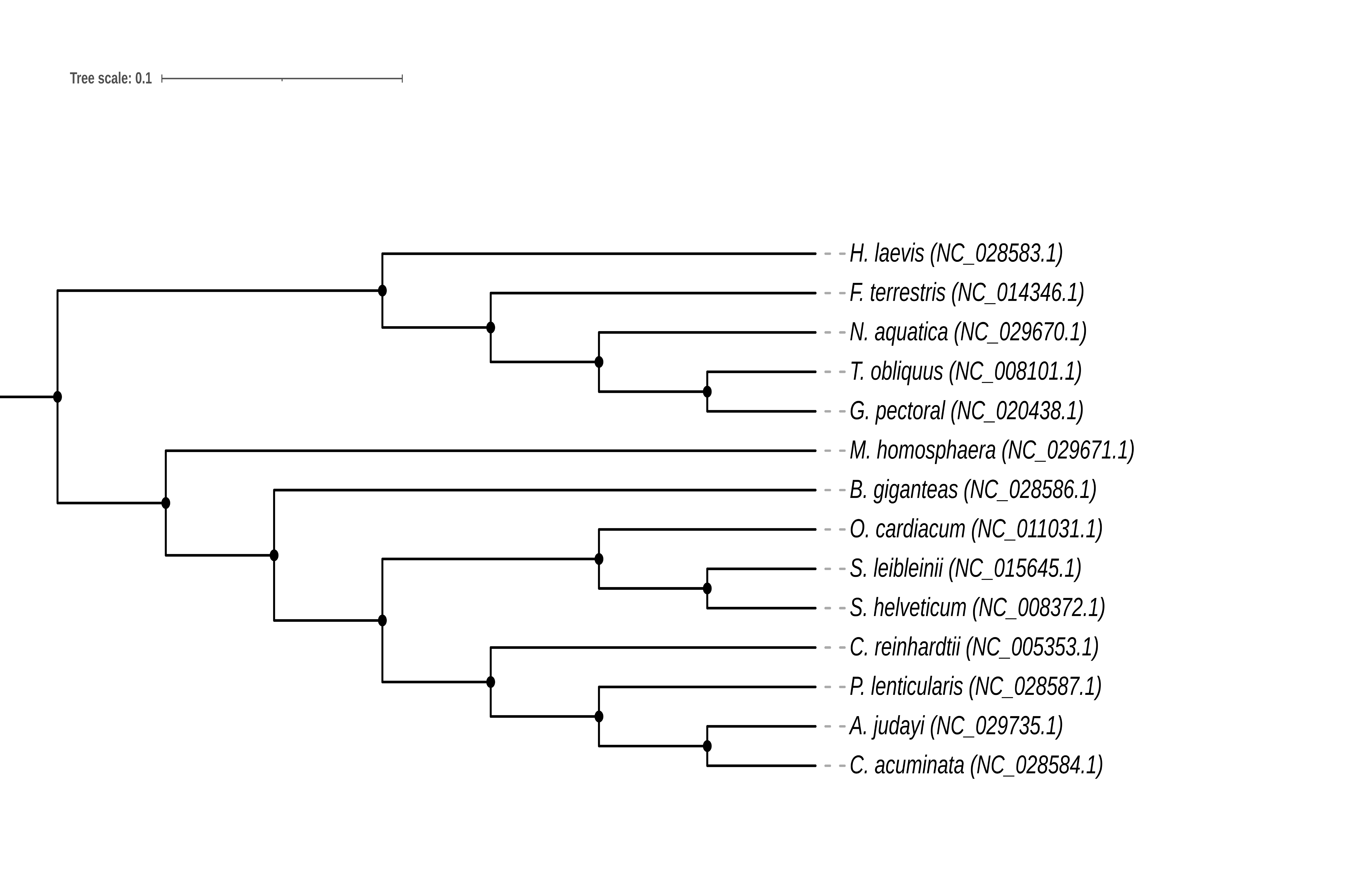

Supplement: Supplemental Information 3 — In the phylogenetic map of chloroplast genome, it was found that the branches of other species were not consistent with the branches of species tree except S. Leibleinii and S. helveticum in Chaetophorales, which indicated that the chloroplast evolution of chlorophyta was not inconsistent with the evolution pattern and direction of species evolution. [file peerj-09-11524-s003.png]
